# Making sense of the carotid body counter‐regulatory response to hypoglycaemia

**DOI:** 10.1113/EP091091

**Published:** 2023-01-05

**Authors:** Aoife D. Slyne, Ken D. O'Halloran

**Affiliations:** ^1^ Department of Physiology School of Medicine College of Medicine & Health University College Cork Cork Ireland

**Keywords:** carotid body, hypoglycaemia, insulin

1

The homeostatic control of blood glucose is critical to life. Contemporary concerns relating to metabolic disorders are primarily focused on the deleterious consequences of abundant energy intake and the resultant long‐term consequences of insulin resistance and hyperglycaemia. However, an acute reduction in blood glucose concentration presents an immediate and substantive stress to the brain, which is avidly countered by neurohumoral responses that serve to restore and protect normoglycaemia.

The carotid bodies are recognised as polymodal sensors that taste the arterial blood perfusing the brain. Whereas there has been a long‐standing interest in the pivotal role of the carotid bodies in oxygen sensing, in recent years it has become evident that carotid body chemoreceptors play a critical role in the reflex response to a range of stressors including the counter‐regulatory response to hypoglycaemia. In this issue of *Experimental Physiology*, Baby et al. (2023) report for the first time the acute effects of insulin‐induced hypoglycaemia on carotid body chemoreceptor activity and cardiorespiratory responsiveness in vivo in a large animal model.

In anaesthetised dogs, afferent neural traffic from both carotid bodies (carotid sinus nerve activity), respiratory flow, heart rate and arterial blood pressure were recorded. Insulin was administered by intracarotid infusion and carotid body and cardiorespiratory responses to intracarotid sodium cyanide (a carotid body stimulant) were determined during normoglycaemia and hypoglycaemia.

Insulin‐mediated hypoglycaemia increased carotid sinus nerve activity and ventilation, which partially offset the hypermetabolic effects of hypoglycaemia. Notably, however, arterial $P_{{\mathrm{CO}}_{2}}$ remained elevated during hypoglycaemia resulting in acidosis, demonstrating that the ventilatory compensation was incomplete. Intriguingly, carotid body and cardiorespiratory responses to sodium cyanide were potentiated in hypoglycaemia compared to normoglycaemia, demonstrating an apparent hypoglycaemia‐dependent sensitisation of the peripheral arterial chemoreceptors to hypoxia. Exploration of the molecular mechanisms of the synergistic crosstalk between the concurrent stressors is clearly worthy of further pursuit given the clinical relevance to respiratory and cardiometabolic disorders.

A key role for the carotid bodies in mediating counter‐regulatory responses to hypoglycaemia has been previously established (Koyama et al., 2000; Thompson et al., 2016; Wehrwein et al., 2010), wherein substantive reductions in corrective responses were observed following carotid body ablation (dog, rat) or hyperoxic ‘silencing’ of the chemoreceptors (human). Interestingly, notwithstanding the central role of the carotid body, counter‐regulatory responses to hypoglycaemia are intact in people with bilateral carotid body resection (Wehrwein et al., 2015) demonstrating the remarkable capacity for plasticity through the employment of reserve or redundant pathways. Nature finds a way!

Baby et al. (2023) observed delayed carotid sinus nerve peak activation in response to intracarotid insulin infusion. The authors highlight that the temporal response is consistent with insulin receptor‐mediated signalling, suggesting a direct action of insulin on the carotid bodies. Of note, insulin receptors are expressed on type 1 glomus cells of the carotid bodies and observations in ex vivo preparations suggest direct insulin sensing by the carotid bodies. However, the argument that insulin directly drives carotid body activation in vivo is obscured by the contemporaneous insulin‐mediated hypoglycaemia, which can partially or even fully account for the excitatory effect. Although controversial, and dependent upon experimental conditions, it is suggested that low glucose can directly activate the carotid bodies. Also, and more plausible, counter‐regulatory hormones released during hypoglycaemia can activate the carotid bodies with strong evidence in rats that adrenaline released during hypoglycaemia is the principal mediator of carotid body excitation in response to insulin‐induced hypoglycaemia (Thompson et al., 2016), critical to hyperpnoea and acid–base balance. Thus, conclusive evidence that insulin directly stimulates the carotid bodies in vivo remains lacking. Assessment of the effects of insulin on carotid body discharge during euglycaemic clamp is required to further probe this important issue relevant to hyperinsulinaemic states.

Making sense of the mediators of carotid body excitation offers tremendous potential for a better understanding of the contributions of the multimodal sensor to physiological homeostasis and delineation of the mechanisms of aberrant signalling that compound a variety of diseases. Although a multi‐pronged, multi‐disciplinary approach is required, fundamental studies in large animal models are key to the development of targeted therapies that translate to treatments for cardiorespiratory and metabolic disorders.

## AUTHOR CONTRIBUTIONS

Both authors have read and approved the final version of this manuscript and agree to be accountable for all aspects of the work in ensuring that questions related to the accuracy or integrity of any part of the work are appropriately investigated and resolved. All persons designated as authors qualify for authorship, and all those who qualify for authorship are listed.

## CONFLICT OF INTEREST

None.

## FUNDING INFORMATION

No funding was received for this work.
